# Immunological Profiling of Leukocyte Subset Proportions and Novel Blood Biomarkers in the Acute Phase of Ocular Sarcoidosis and Vogt–Koyanagi–Harada Disease: An Exploratory Pilot Study

**DOI:** 10.3390/ijms27094139

**Published:** 2026-05-06

**Authors:** Tomohito Sato, Yuki Takenaka, Yoshiaki Nishio, Masataka Ito, Masaru Takeuchi

**Affiliations:** 1 Department of Ophthalmology, National Defense Medical College, Tokorozawa 359-8513, Japan; dr21043@ndmc.ac.jp (T.S.); mii.52yy@gmail.com (Y.T.); cln349@ndmc.ac.jp (Y.N.); 2 Department of Developmental Anatomy and Regenerative Biology, National Defense Medical College, Tokorozawa 359-8513, Japan; masataka@ndmc.ac.jp

**Keywords:** correlation, hierarchical cluster analysis, leukocyte proportion, mass cytometry, ocular sarcoidosis, receiver operating characteristic curve, Vogt-Koyanagi-Harada disease

## Abstract

Aberrant pathogenic immune responses drive autoimmune uveitides; however, comprehensive leukocyte profiling in the conditions remains limited. Here, this exploratory pilot study aimed to elucidate the immunodynamics of ocular sarcoidosis (OS) and Vogt–Koyanagi–Harada disease (VKH) to identify blood diagnostic biomarkers during their acute phases. We performed a prospective observational analysis of ten newly diagnosed, treatment-naïve OS patients and seven VKH patients during their acute phases, along with eight healthy controls (HCs). Mass cytometry was utilized to quantify the proportions of 37 distinct leukocyte subsets. In OS group, the proportion of CD8^+^ naive was lower than in both VKH and control groups. Furthermore, the proportions of CD8^+^ central memory and γδ T cells were decreased compared to HC group. Hierarchical cluster analysis categorized the leukocyte subsets into four principal clusters: Cluster A (Th17-like, monocytes, neutrophils, etc.), Cluster B (Tregs, B cells, NK cells, basophils, etc.), Cluster C (CD8^+^ T cells, Th1-like, Th2-like, DCs, etc.), and Cluster D (CD4^+^ terminal effector, CD8^+^ terminal effector, and CD66b^−^ neutrophils). Compared to HC group, the abundance of Cluster A was relatively high in OS group, and the abundance of cluster B was relatively high in VKH group. In OS group, the proportions of CD8^+^ T cells and CD8^+^ terminal effector correlated negatively with serum ACE and sIL-2R levels. ROC curve analysis estimated that CD4^+^/CD8^+^ ratio (cut-off value: ≥3.46), the proportion of monocytes (≥9.41%), and the decreased proportions of CD3^+^ T cells (≤43.9%) and CD8^+^ T cells (≤10.0%) in peripheral blood may serve as potential blood biomarkers for diagnosing OS. The exploratory pilot study provides a comprehensive and simultaneous data of leukocyte subset proportions in the acute phase of OS and VKH, and our preliminary findings suggest that the proportions of specific leukocyte subsets may represent potential candidates for blood-based biomarkers in the diagnosis of OS.

## 1. Introduction

Uveitis is a sight-threatening intraocular inflammation and a common cause of blindness [1]. It is characterized by heterogeneous clinical manifestations, which collectively account for 10% to 25% of legal blindness worldwide [1,2]. The pathogenesis of uveitis involves complex interactions among diverse genetic, immunological, environmental, and epigenetic factors [3]. Ocular sarcoidosis (OS) and Vogt–Koyanagi–Harada disease (VKH) are the most common non-infectious granulomatous uveitis [4]. The diagnosis of uveitis is often challenging because the condition can be caused by a wide variety of diseases with similar clinical manifestations.

Uveitis is predominantly characterized by an exaggerated immune response to antigens interacting with cluster of differentiation (CD) 4^+^ T cells including T helper 1 (Th1) cells and T helper 17 (Th17) cells, and antigen-presenting cells [5]. The immune response in uveitis can lead to blood–retinal barrier dysfunction, retinitis, choroiditis, and subsequent tissue damage [6,7]. The etiologies of OS and VKH remain a conundrum, and there are no established blood biomarkers for diagnosing these diseases, especially VKH [8,9]. Thus, the diseases are primarily diagnosed based on signs and criteria that are not shared with most types of uveitis [10,11]. In OS, previous research evaluated the ability of promising blood diagnostic biomarkers including angiotensin-converting enzyme (ACE) and soluble interleukin-2 receptor (sIL-2R) [12,13]. However, these markers alone have insufficient detection power for reliable diagnosis of OS [10].

Cytometry by time-of-flight (CyTOF), a form of mass cytometry, represents one of the most powerful tools in immune phenotyping, allowing high-throughput quantification of over 40 parameters with single-cell resolution [14]. Liquid biopsies, which utilize fluid samples such as urine [15], blood [16], aqueous humor [17] and vitreous fluid [18], are attracting attention as a non- or less invasive method of specimen collection [19].

This exploratory pilot study was designed with two primary objectives: first, to characterize the immune profiles of specific leukocyte subsets, providing preliminary insights into the pathophysiology of OS and VKH in the acute phase; and second, to identify potential peripheral blood biomarkers that may assist in the diagnosis of these diseases.

## 2. Results

### 2.1. Profiles of Leukocyte Populations, Phenotypes and Proportions

Leukocyte populations, phenotypes and subset proportions in OS, VKH and HC groups are shown in Table 1. The proportions of CD8^+^ naïve, CD8^+^ central memory and γδ T cells were low in OS group compared to HC group. Furthermore, the proportion of CD8^+^ naïve was lower in OS group than in VKH group. The proportions of the remaining leukocyte subsets did not differ significantly among the three groups.

The immune cell populations and their model definitions determined by the Maxpar Direct Immune Profiling Assay^®^ (MDIPA) are presented in Supplementary Table S1.

### 2.2. Classification of Leukocyte Phenotypes and Proportions by Hierarchical Cluster Analysis

**Figure 1:**
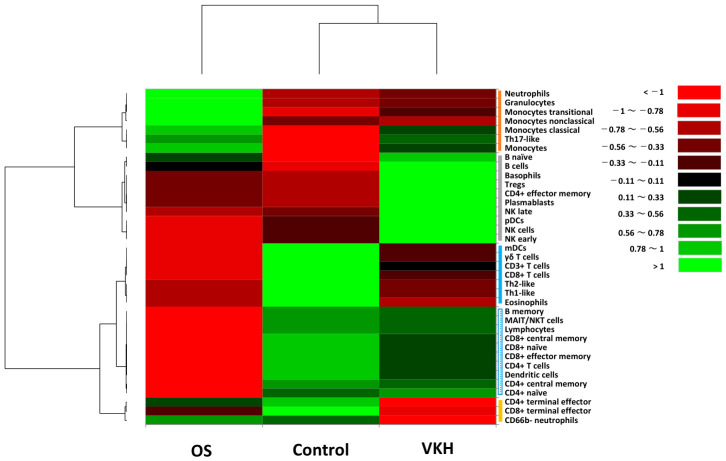
Classification of leukocyte phenotypes and subset proportions by hierarchical cluster analysis. The heatmap illustrates proportions of 37 leukocyte subsets in the acute phase of OS patients, VKH patients and healthy controls. Leukocyte phenotypes are broadly classified into four clusters (indicated as vertical bars on right of the heatmap) based on property similarity: (1) Cluster A [red bar], (2) Cluster B [gray bar], (3) Cluster C [blue bar] and (4) Cluster D [yellow bar]. Cluster C was further divided into Cluster C-1 (blue solid bar) and Cluster C-2 (blue dotted bar). Color scale: low values, red; middle to high values, black to green. Vertical axis indicates leukocyte phenotypes, and horizontal axis shows the three groups.

Hierarchical cluster analysis was performed to classify leukocyte phenotypes and proportions into groups with property similarities called clusters [20]. In this analysis, the leukocyte subsets were categorized into four principal clusters as follows (Figure 1): Cluster A (neutrophils, granulocytes, monocytes transitional, monocytes non-classical, monocytes classical, Th17-like, monocytes), Cluster B (B naïve, B cells, basophils, Tregs, CD4^+^ effector memory, plasmablasts, NK late, pDCs, NK cells, NK early), Cluster C (mDCs, γδ T cells, CD3^+^ T cells, CD8^+^ T cells, Th2-like, Th1-like, eosinophils, B memory, MAIT/NKT cells, lymphocytes, CD8^+^ central memory, CD8^+^ naïve, CD8^+^ effector memory, CD4^+^ T cells, DCs, CD4^+^ central memory, CD4^+^ naïve), and Cluster D (CD4^+^ terminal effector, CD8^+^ terminal effector, CD66b^−^ neutrophils). Cluster C was further divided into Cluster C-1, consisting of mDCs, γδ T cells, CD3^+^ T cells, CD8^+^ T cells, Th2-like, Th1-like, and eosinophils, and Cluster C-2 comprising B memory, MAIT/NKT cells, lymphocytes, CD8^+^ central memory, CD8^+^ naïve, CD8^+^ effector memory, CD4^+^ T cells, DCs, CD4^+^ central memory and CD4^+^ naïve.

The abundance of Cluster A was relatively high in OS group compared to both VKH and HC groups. In contrast, the abundance of cluster B was relatively high in VKH group compared to both OS and HC groups. Regarding Cluster C, the abundance of Cluster C-1 was relatively high in HC group compared to both OS and VKH groups. In contrast, the abundance of Cluster C-2 was relatively low in OS group compared to VKH and HC groups, while it remained comparable between VKH and HC groups. As for the abundance of Cluster D, it was slightly high in HC group compared to OS group, but markedly lower in VKH group than in both OS and HC groups.

In hypothesis-generating summary, our findings suggest that a prominent innate immune response involving neutrophils, monocytes, and Th17-like cells may be an immunological feature in the acute phase of OS. In contrast, the acute phase of VKH might be characterized by a complex hyperimmune response primarily driven by B lymphocyte activation, involving prominent shifts in B cells, basophils, Tregs, and NK cells. Notably, both OS and VKH groups exhibited a common suppression of CD8^+^ T cells, Th1-like, Th2-like, and antigen-presenting mDCs during the acute phase.

### 2.3. Correlation Between Leukocyte Subset Proportions and Serum ACE or sIL-2R Level

**Figure 2:**
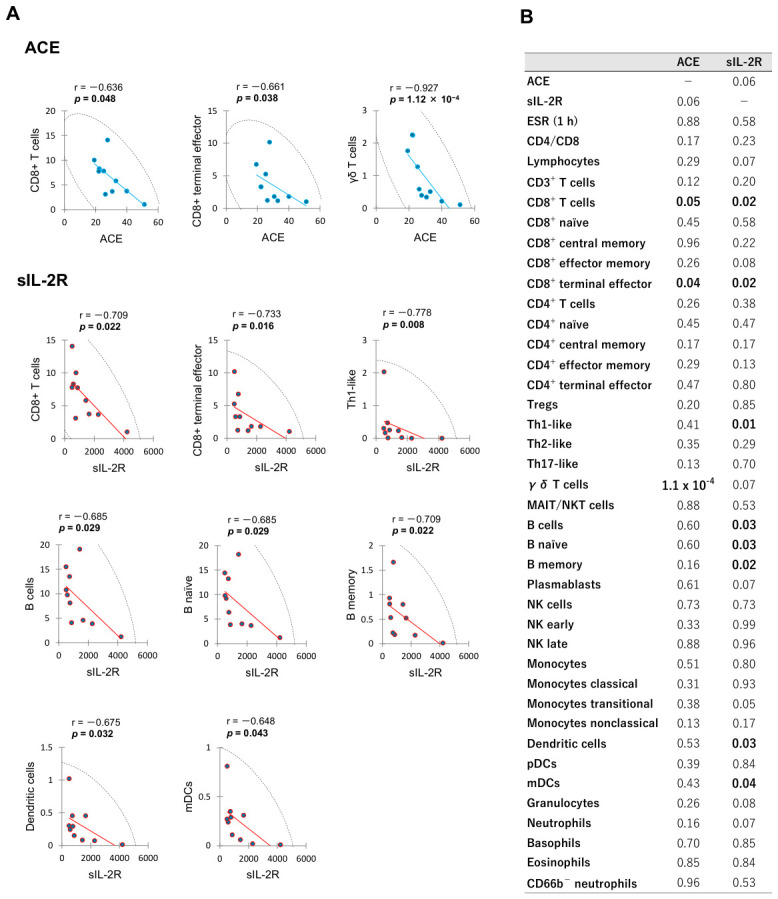
Significant correlations between leukocyte subset proportions and serum ACE or sIL-2R level in the acute phase of OS patients. Spearman’s rank correlations for significant biomarkers are illustrated in (**A**), while a comprehensive matrix of *p* values for all measured variables is presented in (**B**). Proportion of each leukocyte subset (vertical axis) is shown as percentage. Serum ACE and sIL-2R levels (horizontal axis) are expressed in international unit per liter (IU/L) and microgram per milliliter (U/mL), respectively. The color circles in (**A**) are the values for each patient. The line of best fit is shown by the blue or red straight line. Each graph includes 95% confidence ellipse shown by black dotted lines. r; Spearman’s rank correlation coefficient.

The correlation diagrams demonstrating significant relationships between leukocyte subset proportions and serum ACE or sIL-2R level in OS group are presented in Figure 2. In this analysis, negative correlations were detected between serum ACE level and the proportions of CD8^+^ T cells, CD8^+^ terminal effector, and γδ T cells. Serum sIL-2R level showed negative correlation with the proportions of CD8^+^ T cells, CD8^+^ terminal effector, Th1-like, B cells, B naïve, B memory, DCs, and mDCs. A trend of positive correlation was found between serum ACE and sIL-2R levels (*p* = 0.06), although statistically significance was not reached. The *p* value matrix for correlations among all measured variables in OS group is provided in Supplementary Table S2.

**Figure 3:**
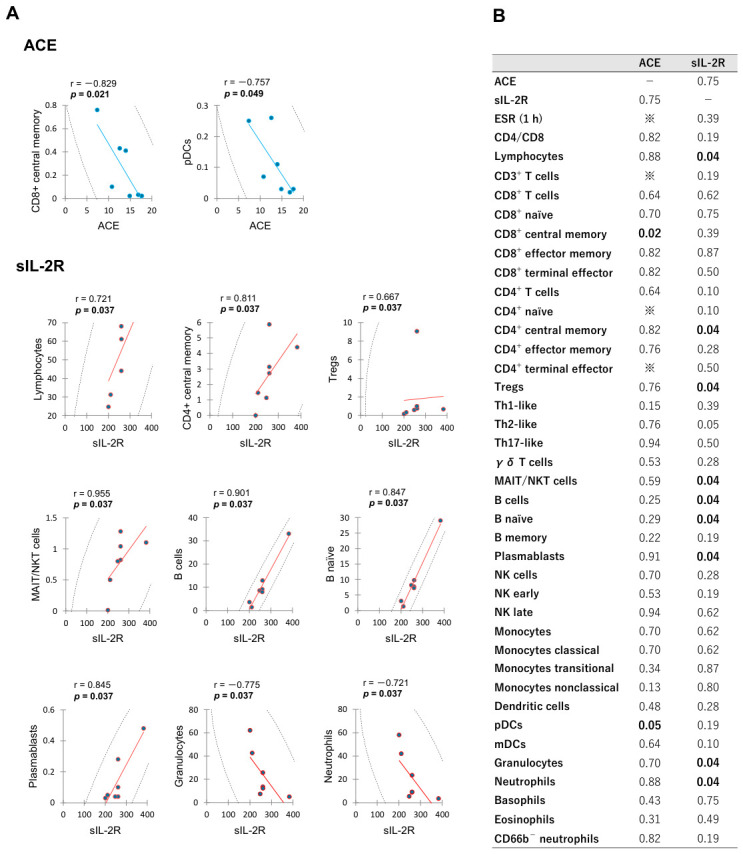
Significant correlations between leukocyte subset proportions and serum ACE or sIL-2R level in the acute phase of VKH patients. Spearman’s rank correlations for significant biomarkers are illustrated in (**A**), while a comprehensive matrix of *p* values for all measured variables is presented in (**B**). ※; Cannot calculate due to correlation coefficient being less than 1 × 10^−10^.

The correlation diagrams showing significant relationships between leukocyte subset proportions and serum ACE or sIL-2R level in VKH group are presented in Figure 3. In this analysis, negative correlations were detected between serum ACE level and the proportions of CD8^+^ central memory and pDCs. Serum sIL-2R level correlated positively with the proportions of lymphocytes, CD4^+^ central memory, Tregs, MAIT/NKT cells, B cells, B naïve, and plasmablasts; and correlated negatively with the proportions of granulocytes and neutrophils. Serum ACE level did not correlate significantly with sIL-2R level (*p* = 0.747). The *p* value matrix for correlations among all measured variables in VKH group is provided in Supplementary Table S3.

The *p* value matrix for correlations among CD4/CD8 ratio and leukocyte subset proportions in HC group is provided in Supplementary Table S4.

### 2.4. Diagnostic Blood Biomarkers for Ocular Sarcoidosis and Vogt–Koyanagi–Harada Disease

Elevated CD4/CD8 ratio (>3.5) in bronchoalveolar lavage (BAL) fluid has been used as a diagnostic criterion for OS [10]. To explore the potential of CD4/CD8 ratio and various leukocyte subset proportions as blood biomarkers for the diagnosis of OS and VKH, receiver operating characteristic (ROC) curve analysis [21] was performed to assess their diagnostic performance.

**Figure 4:**
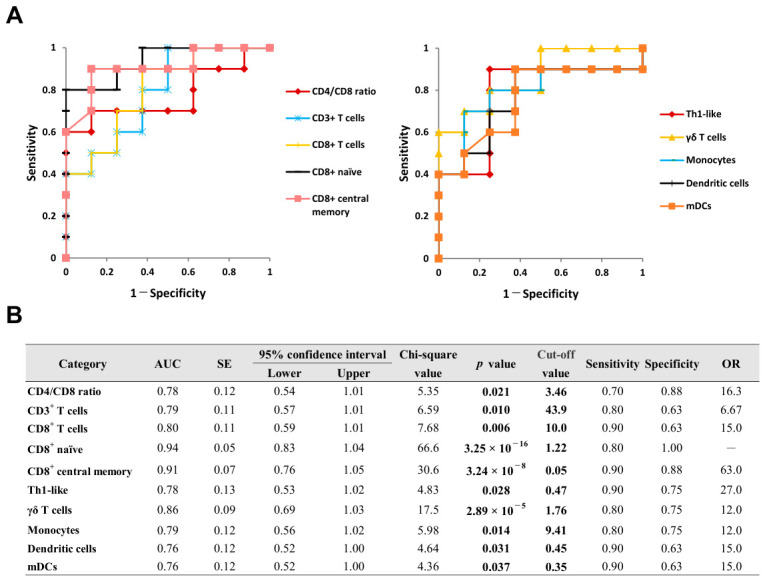
ROC curve analysis of CD4/CD8 ratio and leukocyte subset proportions in peripheral blood for diagnosing OS. ROC curves for significant diagnostic biomarkers are illustrated in (**A**), and their detailed statistical parameters are presented in (**B**). Cut-off value is defined by the point closest to point (0, 1) of the graph. AUC; area under curve, OR; odds ratio, ROC; receiver operating characteristic, SE; standard error.

The ROC curves and detailed diagnostic parameters for the promising blood biomarkers in OS are summarized in Figure 4. CD4/CD8 ratio and the proportions of CD3^+^ T cells, CD8^+^ T cells, CD8^+^ naive, CD8^+^ central memory, Th1-like, γδ T cells, monocytes, DCs, and mDCs were estimated as blood diagnostic biomarkers. The optimal cut-off values for the CD4/CD8 ratio and the proportion of monocytes were 3.46 and 9.41%, respectively, with values equal to or greater than these thresholds considered positive for the diagnosis. In contrast, the optimal cut-off values for the proportions of CD3^+^ T cells, CD8^+^ T cells, CD8^+^ naive, CD8^+^ central memory, Th1-like, γδ T cells, DCs, and mDCs were 43.9%, 10.0%, 1.22%, 0.05%, 0.47%, 1.76%, 0.45%, and 0.35%, respectively. For these parameters, values equal to or less than the thresholds were considered positive for the diagnosis.

**Figure 5:**
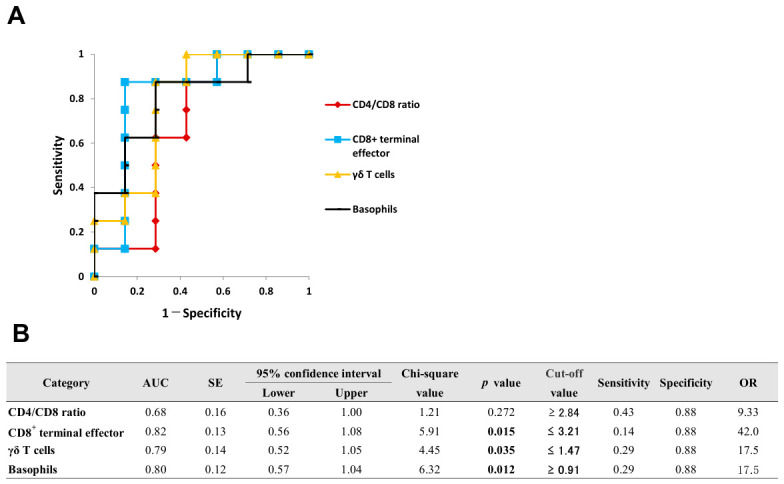
ROC curve analysis of CD4/CD8 ratio and leukocyte subset proportions in peripheral blood for diagnosing VKH. ROC curves for significant diagnostic biomarkers are illustrated in (**A**), and their detailed statistical parameters are presented in (**B**).

Figure 5 illustrates the ROC curves and detailed diagnostic parameters for the promising blood biomarkers in VKH. Although CD4/CD8 ratio was not a significant biomarker (*p* = 0.272), the proportions of CD8^+^ terminal effector T cells, γδ T cells, and basophils were estimated as blood diagnostic biomarkers. The optimal cut-off value for the proportion of basophils monocytes was 0.91%, with values equal to or greater than these thresholds considered positive for the diagnosis. In contrast, the optimal cut-off values for the proportions of CD8^+^ terminal effector T cells and γδ T cells were 3.21% and 1.47%, respectively. For these parameters, values equal to or less than the thresholds were considered positive for the diagnosis.

Based on the reliability [22] and reproducibility of their cut-off values, the CD4/CD8 ratio and the proportions of CD3^+^ T cells, CD8^+^ T cells, and monocytes were selected as promising blood biomarkers for the diagnosis of OS. Conversely, although the proportions of CD8^+^ terminal effector, γδ T cells, and basophils were estimated as promising diagnostic biomarkers for VKH, their optimal cut-off values are considerably low. Therefore, from the perspective of clinical utility and reproducibility, the application of these specific biomarkers in VKH warrants further validation.

## 3. Discussion

This exploratory pilot study utilized mass cytometry to profile leukocyte subset proportions in patients with OS and VKH during the acute phase, in comparison with HCs. By offering a preliminary immunological profile, this study may provide insights into the immunodynamics of these diseases, and explore potential peripheral blood components that warrant further investigation as diagnostic biomarkers for OS and VKH. Our major findings are summarized below. (1) Hierarchical cluster analysis based on property similarity suggests that the primary pathogenesis of OS during the acute phase involves aberrant activation of the innate immune response. In contrast, the immune pathology of VKH during the acute phase may be characterized by a complex hyperimmune response involving B lymphocyte activation. Furthermore, both OS and VKH in the acute phase appear to share a common immunological state characterized by the relative suppression of antigen-presenting cell proportions. (2) In the acute phase of OS, the proportion of CD8^+^ T cells correlates negatively with serum ACE level, a blood biomarker included in the diagnostic criteria for OS [10]. (3) Based on AUC and cut-off values estimated in this preliminary cohort, the CD4/CD8 ratio (≥3.46), the proportion of monocytes (≥9.41%), as well as the decreased proportions of CD3^+^ T cells (≤43.9%), and CD8^+^ T cells (≤10.0%) in peripheral blood may serve as potential candidate biomarkers for the diagnosis of OS, warranting further validation in larger, prospective studies.

Sarcoidosis is an autoimmune disease of unknown etiology characterized by the formation of non-caseating granulomas in various organs [23]. The diagnosis of sarcoidosis is not standardized but is based on three major criteria: (1) a compatible clinical and/or radiological presentation, (2) histological evidence of non-caseating granulomatous inflammation in one or more tissues, and (3) the exclusion of alternative causes of granulomatous disease [23,24]. Specific diagnostic criteria have been established for cutaneous sarcoidosis [25], cardiac sarcoidosis [26], neurological sarcoidosis [27], and OS [10]. In sarcoidosis, the pathophysiological hallmark is the formation of non-caseating epithelioid granulomas, which are distinct aggregates of multinucleated giant cells and epithelioid cells encircled by a rim of CD4^+^ T cells [24]. Less abundant CD8^+^ T cells and B cells are also present in the surrounding rim [28]. The granulomatous inflammation may represent a dysregulated antigenic response to an unknown environmental exposure in genetically susceptible individuals [28].

VKH is a leading cause of uveitis [4], and is a T lymphocyte-mediated autoimmune disease, which targets melanocytes expressing HLA-DR [29]. The diagnosis of VKH is not standardized but is based on five major criteria: (1) no history of penetrating ocular trauma or surgery preceding the initial onset of uveitis, (2) no clinical or laboratory evidence suggestive of other ocular disease entities, (3) bilateral ocular involvement, (4) neurologic/auditory findings (meningismus, tinnitus or cerebrospinal fluid pleocytosis), and (5) integumentary findings (alopecia, poliosis or vitiligo) [11]. With the widespread clinical use of indocyanine green angiography and choroidal enhanced depth imaging, optical coherence tomography, the presence of diffuse choroiditis has been proposed as a diagnostic criterion for initial-onset VKH [9]. The pathogenic cells involved in VKH include memory T cell subsets such as cytotoxic T cells [30], Th1 cells [31], and Th17 cells [31]. Other immune cells including NK cells, B cells and myeloid cells also play a role in the pathology [32,33].

Both OS and VKH are classified as forms of non-infectious granulomatous uveitis; however, their pathophysiology represents fundamentally distinct entities. Previous studies have examined blood biomarkers to explore the primary pathology of these diseases by comparing sarcoidosis patients [34] or VKH patients [16,35] with healthy subjects. However, Shimizu et al. [5] proposed that comparing different uveitis groups, such as sarcoidosis with active uveitis, VKH and Behçet’s disease could provide a clearer understanding of their pathologies than comparisons with healthy subjects. Therefore, through a comparative analysis of OS, VKH and HC groups, we explored the systemic immune profiles based on leukocyte subset proportions in these diseases.

In this exploratory pilot study, the proportions of CD8^+^ naïve, CD8^+^ central memory and γδ T cells in peripheral blood were significantly low in the OS group compared to the HC group. Furthermore, the proportion of CD8^+^ naïve was lower in the OS group than in the VKH group. The hierarchical cluster analysis estimated that the OS group exhibited a relatively high abundance of Cluster A, which was predominantly composed of neutrophils, monocytes and Th17-like, compared to in the HC group (Figure 1). Currently, it is generally recognized that unknown antigens (such as mycobacterial antigens [36] and *Propionibacterium acnes* [37]) and complex immunological interactions in a genetically susceptible host are involved in the pathogenesis of sarcoidosis, and that T cells play a central role in the disease process [38]. CD8^+^ T cells are a crucial component of the adaptive immune system, and play a vital role in immune defense against tumors and intracellular pathogens such as viruses and bacteria [39]. Memory CD8^+^ T cells are classified into various subsets including CD8^+^ naïve and CD8^+^ central memory, depending on the effector function, proliferative capacity, and tissue-homing properties [40]. Recent studies suggest that sarcoidosis is characterized not only by an augmented Th1 immune response, but also potentially dysfunction of regulatory immune cells and immune exhaustion, leading to a failure to clear an antigenic agent [41,42]. In the revised diagnostic criteria for OS [10], lymphopenia has been incorporated as one of the four systemic manifestations. Based on the aforementioned findings and our current results, we suppose that the significant decreases in the proportions of CD8^+^ naive, CD8^+^ central memory and γδ T cells, together with the relative increase in the abundance of Cluster A and relative decreases in the abundance of Cluster C (including lymphocytes, CD8^+^ T cells, Th1-like) may represent the specific immunological manifestations of lymphopenia during the acute phase of OS.

In the pathogenesis of VKH, tyrosinase and tyrosinase-related proteins 1 and 2 from melanocytes have been identified as the primary autoantigens [43]. While Th1 and Th17 polarization is thought to play a major role in the immunopathology of VKH [7,44], a potential role for B lymphocytes has also been proposed [29,33]. Regarding the pathogenesis of VKH using high-dimensional immunological approaches such as CyTOF and single-cell RNA sequencing, several key insights have been reported. Li et al. [33] conducted a comprehensive analysis of peripheral blood mononuclear cells (PBMCs) from VKH patients compared to healthy controls. They reported that the immunological profile of PBMCs in the patients represents a complex mixture of inflammatory, effector, and exhausted states. Furthermore, they identified a novel B cell subset, termed natural killer-like B cells, characterized by the expression of CD19 and CD56, which may promote the differentiation of pathogenic Th1 and Th17 cells. Additionally, Liu et al. [45] analyzed peripheral blood samples from VKH patients compared to healthy controls. They reported that VKH is characterized by a pathological polarization of T cells, shifting from naïve to effector and memory subsets, which is accompanied by an accumulation of monocytes in the blood. In our exploratory pilot study, hierarchical cluster analysis indicated a relatively high abundance of Cluster B (comprising Tregs, B cells, NK cells, and basophils) in VKH group compared to HC group. Additionally, compared to HC group, VKH group exhibited a relatively low abundance of Th1-like and a high abundance of Th17-like. Our results support the hypothesis from previous findings that the immunological features in the acute phase of VKH is characterized by hyperactive immune responses driven by activated T cells and B lymphocytes [29,33], implying a complex immune process involving a Type IV hypersensitivity reaction [46,47].

Correlation analysis is used to determine the interrelationship and interdependence between two variables [48,49]. A strong correlation between two variables enables the mathematical prediction of one variable’s value based on the observed value of the other [50]. In this study, the proportion of CD8^+^ T cells correlates inversely with serum ACE and sIL-2R levels in the acute phase of OS (Figure 2). To date, the relationships among serum ACE levels, sIL-2R levels and leukocyte subset proportions in OS have primarily been investigated in isolation, rather than simultaneously. The potential correlation between these established serum biomarkers and specific leukocyte subset proportions may provide insights into the immune status of OS, potentially offering new perspectives on previously reported findings.

ACE is an acid glycoprotein that converts angiotensin I into angiotensin II [51]. In sarcoidosis, it is mainly produced by activated alveolar macrophages and epithelioid cells within granulomas. Consequently, serum ACE level correlates with granuloma burden and radiological stages II and III [52]. Elevated serum ACE level is observed in approximately 30% to 80% of sarcoidosis patients, with sensitivity and specificity ranging from 22% to 86% and from 54% to 95%, respectively [53]. Soluble IL-2R is the circulating form of the membrane-bound IL-2R, and serves as an established biomarker of disease activity in sarcoidosis [51]. Upon activation, Th1 cells upregulate the expression of IL-2R on their cell surface, and release sIL-2R into circulation [54]. Elevated sIL-2R level could reflect Th1-cell activation in the formation and perpetuation of granulomas of sarcoidosis [6]. A recent study reported that serum sIL-2R level detected sarcoidosis with a sensitivity of 88% and a specificity of 85% [55].

Biomarker is a useful tool for the diagnosis, prognosis, and treatment decisions of many diseases [51]. Liquid biopsies utilizing urine [15], aqueous humor [17] and blood [16] have attracted attention as a non-invasive or minimally invasive method of sampling [19]. Sarcoidosis commonly involves the lungs and intrathoracic lymph nodes [56]. The diagnosis of OS may require invasive methods including BAL fluid collection and transbronchial lung biopsy to obtain histological evidence of non-caseating granulomas [57]. To date, no specific biomarker for OS has been identified [10]. However, CD4/CD8 ratio greater than 3.5 in BAL fluid has been widely employed as a diagnostic biomarker for OS [10]. Furthermore, serum levels of ACE and sIL-2R are considered promising biomarkers for both the diagnosis and prognosis of OS [10,12,13]. In this exploratory pilot study, ROC curve analysis estimated promising blood biomarkers for the diagnosis of OS. In particular, the CD4/CD8 ratio (cut-off value: 3.46, area under the curve [AUC]: 0.78) demonstrated a sensitivity of 70% and a specificity of 88%. Similarly, the proportion of CD8^+^ T cells (cut-off: 10.0%, AUC: 0.80) yielded a sensitivity of 90% and a specificity of 63%. To the best of our knowledge, this is the first study to report that the CD4/CD8 ratio and the proportions of CD3^+^ T cells, CD8^+^ T cells, and monocytes in peripheral blood serve as potential biomarkers for the diagnosis of OS (Figure 4). However, it must be noted that these promising candidates were identified based on a limited sample size. Therefore, the derived results are insufficient for establishing generalized conclusions, and their interpretation must be approached with caution. In addition, to further validate the clinical utility of leukocyte subset proportions as diagnostic biomarkers for OS, it is essential to conduct direct head-to-head comparisons with established markers including serum ACE and sIL-2R. Future research should incorporate these conventional markers alongside the proposed leukocyte subset proportions to evaluate their incremental diagnostic value in clinical practice.

In recent years, several significant diagnostic biomarkers for OS and VKH have been identified using biological specimens beyond peripheral blood. Maruyama et al. [18] reported that in patients with histopathologically verified sarcoidosis, the CD4/CD8 ratio in the vitreous fluid was significantly higher than in the BAL fluid. Chang et al. [15] demonstrated that the combination of acetylglycine and gamma-glutamylalanine in urine could differentiate VKH from healthy controls with an AUC of 0.81 in ROC curve analysis. Wu et al. [17] performed a proteomic analysis of aqueous humor, and reported that transferrin and complement factor B were identified as potential biomarkers for distinguishing between idiopathic uveitis, VKH, and control groups. In the future, an integrated approach combining diverse biomarkers with imaging findings [51] will be essential to achieve more precise diagnoses for OS and VKH.

Our study has several limitations that should be considered when interpreting the results as follows: First, this study is an exploratory pilot study, and consequently, the sample size was insufficient. This limitation is attributed to the rarity of the target diseases, as sarcoidosis has an annual incidence of around 10.1 per million [58], and VKH has an estimated annual prevalence of 15.5 per million and an estimated incidence of 6.5 per million [59]. Therefore, the derived results are insufficient for establishing generalized conclusions, and their interpretation must be approached with caution. Second, the OS group consisted exclusively of patients with presumed OS, while the VKH group was composed entirely of patients with incomplete VKH. Therefore, the findings of this study specifically suppose the immune profiles of these subgroups rather than the entire spectrum of OS and VKH, providing a targeted comparison between presumed OS, incomplete VKH, and HCs. Third, the use of clinically probable diagnoses as the reference standard introduces potential misclassification bias, particularly when immune parameters that may overlap with established diagnostic criteria are used to derive novel biomarkers. Therefore, our findings should be interpreted with caution. Future large-scale clinical studies incorporating histopathologically confirmed sarcoidosis cases are warranted to rigorously evaluate the diagnostic utility and validity of leukocyte subset proportions as biomarkers for OS. Fourth, blood tests including serum ACE and sIL-2R were performed only in patients with OS and VKH, and these data were not available for HCs. This constraint stems from the ethics committee’s mandate to minimize blood sampling volumes and ensure minimally invasive procedures, which restricted the volume of blood collected from HCs to the amount strictly required for CyTOF. Fifth, this study population was composed exclusively of Japanese individuals. Therefore, our findings may not be directly generalizable to other ethnic groups, such as Caucasian, Hispanic, or African American populations. Furthermore, potential confounding factors including comorbidities and the influence of coronavirus disease 2019 (COVID-19) infection or vaccination on systemic immunity were not evaluated. These factors may significantly modulate leukocyte subset proportions, and overall performance of the biomarkers. Sixth, the exact duration from disease onset to initial consultation was not precisely determined. The observed immune profiles may reflect a combination of disease-specific inflammation and generalized inflammatory responses. This possibility should be carefully considered when interpreting the data. Seventh, due to the inherent technical characteristics of the MDIPA platform, our results are limited to the proportions of leukocyte subsets, and absolute cell counts were not determined. It is important to note that proportional data may obscure biologically significant shifts in immune status, particularly in cases where total leukocyte counts vary between individuals or groups. Eighth, multiple comparisons across numerous leukocyte subsets were performed without statistical correction (Bonferroni correction, etc.). Given this approach and the small cohort size, the findings of this study should be interpreted as hypothesis-generating. Finally, the hierarchical cluster analysis was an unsupervised analysis, which inherently allowed for some interpretive freedom. Consequently, interpretations based on unsupervised clustering and qualitative heatmap inspection in small cohorts are tempered, and mechanistic explanations are presented as speculative.

## 4. Materials and Methods

### 4.1. Subjects and Diagnosis

This prospective observational study included 10 patients with newly diagnosed, treatment-naïve OS and 7 patients with newly diagnosed treatment-naïve VKH in the acute phase, along with 8 HCs recruited from the hospital staff. The study was conducted at the National Defense Medical College Hospital between 1 November 2020 and 31 January 2024.

The inclusion criteria were defined as follows: (1) no history of systemic anti-inflammatory medications including corticosteroids and immunosuppressive drugs; (2) no history of COVID-19 infection and its vaccination within 6 months before the enrollment; (3) no history of intraocular inflammatory diseases such as retinal artery occlusion, retinal vein occlusion, age-related macular degeneration, ocular tumor, uveitis, endophthalmitis, and dialysis therapy for renal failure; (4) no history of previous pars plana vitrectomy, ocular trauma, and prior intravitreal therapies including corticosteroids and anti-vascular endothelial growth factor agents; and (5) no history of cataract surgery performed within 6 months before the enrollment. The exclusion criteria were defined as follows: (1) inability to rule out other potential causes of granulomatous uveitis; and (2) presence of clinical or laboratory evidence suggestive of other ocular disease entities.

All patients with OS and VKH underwent a standardized diagnostic workup (including chest X-ray, chest electrocardiogram, blood tests with serum ACE and sIL-2R levels, urinalysis, and cerebrospinal fluid analysis for VKH) prior to CyTOF, and blood tests evaluated in this study. The definitive diagnosis for each patient was reached by consensus during ophthalmic clinical conferences. Following the diagnosis and the obtaining of written informed consent, peripheral blood samples for CyTOF and the blood tests were collected immediately and simultaneously, before the initiation of any systemic treatment. OS was diagnosed according to the diagnostic criteria of International Workshop on Ocular Sarcoidosis revised in 2019 [10], and VKH was diagnosed based on the international diagnostic criteria [11]. Three uveitis specialists (members of the Japanese Ocular Inflammation Society) reviewed the clinical findings of patients with OS or VKH, and confirmed the diagnoses and disease classifications. The data of leukocyte subset proportions were not used in the initial differential diagnosis, ensuring that our results are derived solely from cases confirmed through independent clinical and radiological criteria.

The study protocol was reviewed and approved by the Ethics Committee of National Defense Medical College, and the procedures conformed to the tenets of the Declaration of Helsinki. Written informed consent was obtained from all the participants before the enrollment.

### 4.2. Demographics and Clinical Data

The demographics and clinical data of OS, VKH and HC groups are shown in Table 2. All 10 OS patients were classified as presumed OS according to the diagnostic criteria [10]. Among them, all patients fulfilled the clinical diagnostic criteria for sarcoidosis, and were classified as highly probable, presenting with both uveitis and bilateral hilar adenopathy [24]. All 7 VKH patients were diagnosed with incomplete VKH based on diagnostic criteria [11]. The male-to-female ratio in OS group was approximately 1:3, which is consistent with the findings of a previous study [60]. There were no significant differences in age and male-to-female ratio among the three groups. Erythrocyte sedimentation rate level was elevated above the upper limit of the normal range both in OS and VKH groups. In OS group, aspartate aminotransferase, ACE and sIL-2R levels also exceeded the upper limits.

**Table 1:** Leukocyte populations, phenotypes and subset proportions in the acute phase of ocular sarcoidosis patients, Vogt–Koyanagi–Harada disease patients and healthy controls.

Populations	Model Phenotypes	OS	VKH	Control	*p* Value			
*n*		10	7	8	Kruskal−Wallis	Steel−Dwass		
						Control vs. OS	Control vs. VKH	OS vs. VKH
Intact live cells (%)		100	100	100				
Lymphocytes	CD3 T cells + B cells + NK cells + plasmablasts	46.1 ± 19.5	55.1 ± 22.0	57.6 ± 16.9	0.635			
CD3^+^ T cells	CD8 T cells + CD4 T cells + γδ T cells + MAIT/NKT cells	29.6 ± 14.0	35.3 ± 13.1	42.0 ± 11.0	0.154			
CD8^+^ T cells	CD3+CD66b- CD19- CD8+ CD4- CD14- CD161- TCRγδ- CD123- CD11c-	6.53 ± 3.85	8.36 ± 5.63	12.7 ± 6.46	0.081			
*Naïve*	CD8 T cells + CD45RA+ CCR7+ CD27+	1.22 ± 1.17	4.12 ± 5.11	5.24 ± 4.21	**0.002**	**0.005**	0.478	**0.030**
*Central memory*	CD8 T cells + CD45RA- CCR7+ CD27+	0.04 ± 0.66	0.25 ± 0.29	0.34 ± 0.53	**0.012**	**0.010**	0.830	0.129
*Effector memory*	CD8 T cells + CCR7- CD27+	1.70 ± 1.11	1.76 ± 1.11	1.78 ± 1.05	0.886			
*Terminal effector*	CD8 T cells + CCR7- CD27-	3.57 ± 3.00	2.24 ± 2.32	5.59 ± 5.49	0.091			
CD4^+^ T cells	CD66b-CD3+CD8-CD4+CD14-TCRgd- CD11c-	21.6 ± 11.2	24.3 ± 9.56	25.3 ± 5.20	0.903			
*Naïve*	CD4 T cells + CD45RA+ CCR7+ CD27+	12.3 ± 9.15	15.5 ± 6.65	15.3 ± 5.30	0.607			
*Central memory*	CD4 T cells + CD45RA- CCR7+ CD27+	1.79 ± 1.11	2.67 ± 2.02	2.70 ± 2.40	0.723			
*Effector memory*	CD4 T cells + CD45RA- CCR7- CD27+	3.48 ± 2.06	4.43 ± 2.21	3.33 ± 2.51	0.817			
	CD4 T cells + CD45RA- CCR7- CD27-	4.22 ± 3.23	1.75 ± 0.49	5.04 ± 4.55	0.232			
**Treg cells**	CD4 T cells + CD25+ CD127- CCR4+	0.63 ± 0.39	1.78 ± 3.21	0.40 ± 0.28	0.290			
**Th1-like cells**	CD4 T cells + CXCR3+ CCR6- CXCR5- CCR4-	0.35 ± 0.61	0.38 ± 0.25	0.84 ± 0.59	0.078			
**Th2-like cells**	CD4 T cells + CXCR3- CCR6- CXCR5- CCR4+	1.34 ± 0.83	1.36 ± 0.81	1.65 ± 0.72	0.814			
**Th17-like cells**	CD4 T cells + CXCR3- CCR6+ CXCR5- CCR4+	2.58 ± 2.24	2.41 ± 1.77	1.45 ± 0.88	0.431			
**γδ T cells**	CD66b- CD3+ CD8dim,- CD4- CD14- TCR gd dim,+	0.96 ± 0.84	1.79 ± 1.95	3.14 ± 2.07	**0.023**	**0.027**	0.152	0.591
CD4^-^ T Cells								
**MAIT/NKT** **cells**	CD66b-CD3+CD4-CD14-CD161+ TCRgd- CD28+ CD16-	0.45 ± 0.43	0.79 ± 0.43	0.86 ± 1.25	0.289			
B cells	CD3- CD14- CD56- CD16 dim,- CD19+ CD20+ HLA-DR dim,+	9.06 ± 5.76	10.9 ± 10.4	7.71 ± 4.60	0.913			
*Naïve*	B cells + CD27-	8.39 ± 5.54	9.45 ± 9.12	6.16 ± 3.74	0.786			
*Memory*	B cells + CD27+	0.58 ± 0.49	1.35 ± 1.33	1.48 ± 1.13	0.235			
*Plasmablasts*	CD3- CD14- CD16-,dim CD66b- CD20- CD19+ CD56- CD38++ CD27+	0.08 ± 0.05	0.15 ± 0.17	0.07 ± 0.04	0.807			
NK cells	CD14- CD3- CD123- CD66b- CD45RA+ CD56 dim,+	7.43 ± 3.20	8.90 ± 4.40	7.85 ± 5.97	0.762			
*Early*	NK cells + CD57-	2.37 ± 1.08	3.65 ± 1.77	2.90 ± 2.84	0.241			
*Late*	NK cells + CD57+	5.06 ± 2.31	5.25 ± 2.95	5.07 ± 3.79	0.847			
Monocytes	CD3- CD19- CD56- CD66b- HLA-DR+ CD11c+	11.2 ± 4.62	10.0 ± 2.89	8.02 ± 1.63	0.091			
**Classical**	Monocytes + CD14+ CD38+	9.65 ± 4.00	8.99 ± 2.55	7.03 ± 1.80	0.208			
**Transitional**	Monocytes + CD14 dim CD38 dim	1.02 ± 0.71	0.80 ± 0.31	0.68 ± 0.24	0.483			
**Nonclassical**	Monocytes + CD14- CD38-	0.57 ± 0.50	0.25 ± 0.16	0.30 ± 0.24	0.397			
Dendritic cells	pDCs+ mDCs	0.31 ± 0.29	0.42 ± 0.32	0.47 ± 0.20	0.221			
**Plasmacytoid DCs**	CD3- CD19- CD14- CD20- CD66b- HLA-DR dim,+ CD11c- CD123+	0.06 ± 0.07	0.11 ± 0.10	0.08 ± 0.07	0.474			
**Myeloid DCs**	CD3- CD19- CD14- CD20- HLA-DR dim,+ CD11c dim,+ CD123- CD16 dim,- CD38 dim,+ CD294- HLA-D	0.25 ± 0.23	0.31 ± 0.23	0.40 ± 0.15	0.192			
Granulocytes	Neutrophils + basophils + eosinophils + CD66b- neutrophils	32.8 ± 20.3	24.0 ± 21.2	22.0 ± 15.7	0.523			
**Neutrophils**	CD66b dim,+ CD16+ HLA-DR-	29.8 ± 19.1	21.6 ± 21.0	19.3 ± 13.2	0.506			
**Basophils**	HLA-DR- CD66b- CD123 dim,+ CD38+ CD294+	0.59 ± 0.60	1.20 ± 0.88	0.47 ± 0.45	0.122			
**Eosinophils**	CD14- CD3- CD19- HLA-DR- CD294+ CD66b dim,+	0.13 ± 0.29	0.14 ± 0.14	0.86 ± 1.70	0.309			
**CD66b-** **neutrophils**	CD3- CD19- CD66b- CD56- HLA-DR- CD123- CD45-	2.23 ± 2.87	1.04 ± 0.94	2.01 ± 3.54	0.556			

Leukocyte phenotypes were defined based on the classification framework by Bagwell et al. [14]. Bold font denotes classification of leukocyte subsets based on differentiation and function. Italic font denotes classification based on maturity stage. CD; cluster of differentiation, DCs; dendritic cells, HLA; human leukocyte antigen, MAIT; mucosal-associated invariant T, mDCs; myeloid DCs, NK; natural killer, NKT; natural killer T, pDCs; plasmacytoid DCs, Th; T helper, Tregs; regulatory T cells.

**Table 2:** Demographics and clinical data of ocular sarcoidosis patients, Vogt–Koyanagi–Harada disease patients, and healthy controls.

**Category**	**OS**	**VKH**	**Control**	*p* Value	**Unit**	**Reference Range**
***n***	**10**	**7**	**8**			
	**Mean ± SD**	**Mean ± SD**	**Mean ± SD**			
Age	53.7 ± 17.9	52.7 ± 13.1	38.5 ± 10.6	0.110	year	
Gender (M/F)	2/8	¾	4/4	0.380		
**Hematology**						
RBC	4.81 ± 0.46	4.71 ± 0.55	—	—	×10^6/^μL	3.86~4.92
Hb	13.7 ± 1.05	14.3 ± 1.25	—	—	g/dL	13.7~16.8
Ht	41.9 ± 2.71	43.7 ± 3.47	—	—	%	35.1~44.4
WBC	5.43 ± 1.81	7.56 ± 1.21	—	—	×10^3^/μL	3.3~8.6
Neutrophil	64.6 ± 8.80	68.9 ± 9.92	—	—	%	38.5~80.5
Lymphocyte	26.0 ± 8.83	23.6 ± 7.72	—	—	%	16.5~49.5
MONO	6.84 ± 1.96	4.87 ± 1.70	—	—	%	2.0~10.0
EOSINO	2.14 ± 1.31	2.21 ± 3.01	—	—	%	0~8.5
BASO	0.44 ± 0.30	0.39 ± 0.15	—	—	%	0~2.5
Platelet	262.1 ± 64.3	278.6 ± 39.7	—	—	×10^3^/μL	158~348
ESR (1 h)	31.1 ± 19.6	18.4 ± 10.5	—	—	mm	3~11
**Biochemistry**						
AST	29.4 ± 17.9	18.3 ± 3.59	—	—	U/L	13~30
ALT	33.3 ± 40.2	15.3 ± 2.56	—	—	U/L	7~23
LDH	208.4 ± 28.9	202.4 ± 54.1	—	—	U/L	124~222
ACE	29.8 ± 9.65	13.5 ± 3.58	—	—	IU/L	7.7~29.4
**Endocrine**						
sIL-2R	1355.2 ± 1161.9	260.9 ± 72.7	—	—	U/mL	157~474
**Immunity**						
CRP	0.23 ± 0.39	0.18 ± 0.45	—	—	mg/dL	≤0.3
IgG	1439.7 ± 336.0	1291.0 ± 173.5	—	—	mg/dL	861~1747
IgA	260.8 ± 81.5	178.4 ± 29.8	—	—	mg/dL	93~393
IgM	110.9 ± 59.5	111.4 ± 54.8	—	—	mg/dL	50~269

Ages were compared among OS, VKH and control groups using Kruskal−Wallis test. Gender was compared among the three groups by Fisher’s exact test. ACE; angiotensin-converting enzyme, ALT; aspartate aminotransferase, AST; alanine transaminase, BASO; basophilic cytoplasmic inclusion body, CRP; C-reactive protein, EOSINO; eosinocyte, ESR; erythrocyte sedimentation rate, F; female, Hb; hemoglobin, Ht; hematocrit, Ig; immunoglobulin, LDH; lactic acid dehydrogenase, M; male, MONO; monocyte, OS; ocular sarcoidosis, RBC; red blood cell, SD; standard deviation, sIL-2R; soluble interleukin-2 receptor, VKH; Vogt–Koyanagi–Harada disease, WBC; white blood cell.

### 4.3. Leukocyte Phenotypes and Proportions

Peripheral blood samples were collected in heparinized vacutainer tubes. The samples were transported to St. Luke’s SRL Advanced Clinical Research Center (Tokyo, Japan) for measurements, data processing and analysis. The phenotypes and proportions of leukocyte subsets were automatically calculated by CyTOF (HeliosTM; Fluidigm, South San Francisco, CA, USA) and MDIPA (Fluidigm) [14]. The leukocytes were classified based on differentiation and function as well as maturation stage into 37 types of immune cells as follows: lymphocytes, CD3^+^ T cells, CD8^+^ T cells, CD8^+^ naïve, CD8^+^ central memory, CD8^+^ effector memory, CD8^+^ terminal effector, CD4^+^ T cells, CD4^+^ naïve, CD4^+^ central memory, CD4^+^ effector memory, CD4^+^ terminal effector, regulatory T cells (Tregs), Th1-like, T helper 2 (Th2)-like, Th17-like, gamma delta (γδ) T cells, mucosal-associated invariant T (MAIT)/natural killer T (NKT) cells, B cells, B naïve, B memory, plasmablasts, natural killer (NK) cells, NK early, NK late, monocytes, monocytes classical, monocytes transitional, monocytes non-classical, dendritic cells (DCs), plasmacytoid DCs (pDCs), myeloid DCs (mDCs), granulocytes, neutrophils, basophils, eosinophils, and CD66b^−^ neutrophils.

Whole blood staining, sample acquisition and data normalization were performed according to the manufacturer’s instructions [14]. The flow cytometry standard files generated by HeliosTM were analyzed by Maxpar Pathsetter, an automated analysis system powered by GemStoneTM 3.0.15 (Verity Software House, Topsham, ME, USA) [14].

### 4.4. Statistical Analysis

Statistical analyses were performed using the statistic add-in software for Excel (BellCurve for Excel^®^, SSRI Co., Ltd., Tokyo, Japan, version 4.10; and XLSTAT^®^, Addinsoft Company, Paris, France, version 2025.2.0). Data are expressed as mean ± standard deviation (SD) [61]. Fisher’s exact test was used to compare categorical variables. Multiple comparisons for unpaired, non-parametric data were performed using the Kruskal–Wallis test, followed by the Steel–Dwass test. Spearman’s rank correlation was employed to assess the correlation between unpaired, non-parametric variables. Hierarchical cluster analysis was performed using Ward’s method with Euclidean distance as the distance metric [16]. The mean value was used as the assigned value for the analysis. A two-tailed *p* value less than 0.05 was considered to be statistically significant.

We performed a post hoc power analysis using clinical data of the present study. We calculated an effect size (Cohen’s d) for the proportion of CD8^+^ naive in peripheral blood, which is a representative immune cell proportion with significant difference between OS and control groups (OS: *n* = 10, 1.22 [mean] ± 1.17 [SD], controls: *n* = 8, 5.25 ± 4.21). The effect size for the CD8^+^ naive between these groups was 1.46. To confirm significant differences in the proportion of CD8^+^ naive with a statistical power of 0.80 [62], the sample size required would be 8.3 for two-tailed comparison between the groups. Therefore, based on the aforementioned calculated sample size, we attempted to recruit approximately eight cases for OS, VKH and control groups.

## 5. Conclusions

This exploratory pilot study was designed to perform comprehensive immunological profiling of leukocyte subset proportions in the acute phase of OS and VKH using CyTOF. Hierarchical cluster analysis based on property similarities among leukocyte subset proportions supposed that aberrant activation of the innate immune response constitutes the primary pathogenesis in the acute phase of OS, whereas a complex hyperimmune response driven by B lymphocyte activation is a prominent feature in the acute phase of VKH. Furthermore, the proportion of CD8^+^ T cells in peripheral blood correlated negatively with serum ACE level, an established diagnostic biomarker for OS. Our findings propose that specific leukocyte subset proportions including CD4/CD8 ratio and the proportion of CD8^+^ T cells serve as promising blood-based biomarkers for the diagnosis of OS. Future research focusing on high-performance diagnostic panels which integrating established markers such as serum ACE and sIL-2R levels with these promising leukocyte subset proportions, could provide a valuable, minimally invasive liquid biopsy tool for the accurate diagnosis of OS.

## Data Availability

The original contributions presented in this study are included in the article. Further inquiries can be directed to the corresponding author.

## Supplementary Materials

The following supporting information can be downloaded at: https://www.mdpi.com/article/10.3390/ijms27094139/s1.

## Abbreviations

The following abbreviations are used in this manuscript

ACE	angiotensin-converting enzyme
ALT	aspartate aminotransferase
AST	alanine transaminase
AUC	area under the curve
BAL	bronchoalveolar lavage
BASO	basophil
CD	cluster of differentiation
COVID-19	coronavirus disease 2019
CRP	C-reactive protein
CyTOF	cytometry by time-of-flight
DCs	dendritic cells
EOSINO	eosinocyte
ESR	erythrocyte sedimentation rate
F	female
Hb	hemoglobin
HLA	human leukocyte antigen
Ht	hematocrit
Ig	immunoglobulin
IFN-γ	interferon-gamma
LDH	lactic acid dehydrogenase
M	male
MAIT	mucosal-associated invariant T
mDCs	myeloid DCs
MONO	monocyte
NK	natural killer
NKT	natural killer T
OR	odds ratio
OS	ocular sarcoidosis
PBMCs	peripheral blood mononuclear cells
PCA	principal component analysis
pDCs	plasmacytoid DCs
RBC	red blood cell
ROC	receiver operating characteristic curve
sIL-2R	soluble interleukin-2 receptor
SD	standard deviation
SE	standard error
Th1	T helper 1
Th2	T helper 2
Th17	T helper 17
Tregs	regulatory T cells
WBC	white blood cell
VKH	Vogt–Koyanagi–Harada disease
γδ	gamma delta
