# Supplementary material for: Immunological Profiling of Leukocyte Subset Proportions and Novel Blood Biomarkers in the Acute Phase of Ocular Sarcoidosis and Vogt–Koyanagi–Harada Disease: An Exploratory Pilot Study
Source: Int J Mol Sci. 2026 May 6;27(9):4139. doi: 10.3390/ijms27094139 (PMC13164564; doi:10.3390/ijms27094139)
Supplement: Supplementary file 1 [file ijms-27-04139-s001.zip › ijms-4186143-supplementary.pdf]

Supplementary Table S1. Immune cell populations and model definitions determined by the Maxpar Direct Immune Profiling Assay

Populations	Model phenotypes
Intact live cells (%)	
Lymphocytes	CD3 T cells + B cells + NK cells + plasmablasts
$CD3^+$ T cells	CD8 T cells + CD4 T cells + γδ T cells + MAIT/NKT cells
$CD8^+$ T cells	$CD3^+$ $CD66b^-$ $CD19^-$ $CD8^+$ $CD4^-$ $CD14^-$ $CD161^-$ $TCRgd^-$ $CD123^-$ $CD11c^-$
Naïve	CD8 T cells + $CD45RA^+$ $CCR7^+$ $CD27^+$
Central memory	CD8 T cells + $CD45RA^-$ $CCR7^+$ $CD27^+$
Effector memory	CD8 T cells + $CCR7^-$ $CD27^+$
Terminal effector	CD8 T cells + $CCR7^-$ $CD27^-$
$CD4^+$ T cells	$CD66b^-$ $CD3^+$ $CD8^-$ $CD4^+$ $CD14^-$ $TCRgd^-$ $CD11c^-$
Naïve	CD4 T cells + $CD45RA^+$ $CCR7^+$ $CD27^+$
Central memory	CD4 T cells + $CD45RA^-$ $CCR7^+$ $CD27^+$
Effector memory	CD4 T cells + $CD45RA^-$ $CCR7^-$ $CD27^+$
Terminal effector	CD4 T cells + $CD45RA^-$ $CCR7^-$ $CD27^-$
**Treg cells**	CD4 T cells + $CD25^+$ $CD127^-$ $CCR4^+$
**Th1-like cells**	CD4 T cells + $CXCR3^+$ $CCR6^-$ $CXCR5^-$ $CCR4^-$
**Th2-like cells**	CD4 T cells + $CXCR3^-$ $CCR6^-$ $CXCR5^-$ $CCR4^+$
**Th17-like cells**	CD4 T cells + $CXCR3^-$ $CCR6^+$ $CXCR5^-$ $CCR4^+$
**γδ T cells**	$CD66b^-$ $CD3^+$ CD8dim,$^-$ $CD4^-$ $CD14^-$ TCRγδ dim,$^+$
$CD4^-$ T Cells	
**MAIT/NKT cells**	$CD66b^-$ $CD3^+$ $CD4^-$ $CD14^-$ $CD161^+$ $TCRgd^-$ $CD28^+$ $CD16^-$
B cells	$CD3^-$ $CD14^-$ $CD56^-$ CD16 dim,$^-$ $CD19^+$ $CD20^+$ HLA-DR dim,$^+$
Naïve	B cells + $CD27^-$
Memory	B cells + $CD27^+$
Plasmablasts	$CD3^-$ $CD14^-$ CD16 dim,$^-$ $CD66b^-$ $CD20^-$ $CD19^+$ $CD56^-$ $CD38^{++}$ $CD27^+$
NK cells	$CD14^-$ $CD3^-$ $CD123^-$ $CD66b^-$ $CD45RA^+$ CD56 dim,$^+$
Early	NK cells + $CD57^-$
Late	NK cells + $CD57^+$
Monocytes	$CD3^-$ $CD19^-$ $CD56^-$ $CD66b^-$ $HLA\text{-}DR^+$ $CD11c^+$
**Classical**	Monocytes + $CD14^+$ $CD38^+$
**Transitional**	Monocytes + CD14 dim CD38 dim
**Nonclassical**	Monocytes + $CD14^-$ $CD38^-$
Dendritic cells	pDCs+ mDCs
**Plasmacytoid DCs**	$CD3^-$ $CD19^-$ $CD14^-$ $CD20^-$ $CD66b^-$ HLA-DR dim,$^+$ $CD11c^-$ $CD123^+$
**Myeloid DCs**	$CD3^-$ $CD19^-$ $CD14^-$ $CD20^-$ HLA-DR dim,$^+$ CD11c dim,$^+$ $CD123^-$ CD16 dim,$^-$ CD38 dim,$^+$ CD294- HLA-D
Granulocytes	Neutrophils + basophils + eosinophils + $CD66b^-$ neutrophils
**Neutrophils**	CD66b dim,$^+$ $CD16^+$ $HLA\text{-}DR^-$
**Basophils**	$HLA\text{-}DR^-$ $CD66b^-$ CD123 dim,$^+$ $CD38^+$ $CD294^+$
**Eosinophils**	$CD14^-$ $CD3^-$ $CD19^-$ $HLA\text{-}DR^-$ $CD294^+$ CD66b dim,$^+$
**$CD66b^-$ neutrophils**	$CD3^-$ $CD19^-$ $CD66b^-$ $CD56^-$ $HLA\text{-}DR^-$ $CD123^-$ $CD45^-$

Leukocyte phenotypes were defined based on the classification framework by Bagwell et al. [14]. Nomenclature such as dim,+ denotes that dim to positive events is selected. CD; cluster of differentiation, DCs; dendritic cells, HLA; human leukocyte antigen, MAIT; mucosal associated invariant T, mDCs; myeloid DCs, NK; natural killer, NKT; natural killer T, pDCs; plasmacytoid DCs, Th; T helper, Tregs; regulatory T cells.

Supplementary Table S2. Correlation matrix of serum ACE level, serum sIL-2R level, ESR, CD4/CD8 ratio and leukocyte subset proportions in the acute phase of ocular sarcoidosis patients.

		1	2	3	4	5	6	7	8	9	10	11	12	13	14	15	16	17	18	19	20
1	ACE	–	0.060	0.881	0.174	0.293	0.117	**0.048**	0.446	0.959	0.260	**0.038**	0.260	0.446	0.174	0.293	0.467	0.199	0.413	0.347	0.126
2	sIL-2R		–	0.580	0.229	0.074	0.200	**0.022**	0.580	0.216	0.082	**0.016**	0.385	0.467	0.174	0.128	0.803	0.854	**0.008**	0.293	0.700
3	ESR (1 h)			–	0.855	0.907	0.489	0.276	0.934	0.263	0.556	0.200	0.347	0.627	0.425	0.803	0.987	0.789	0.626	0.117	0.498
4	CD4/CD8				–	0.881	0.855	0.090	0.907	0.434	0.556	**0.043**	0.385	0.098	0.244	0.751	0.108	0.544	0.626	0.803	0.815
5	Lymphocytes					–	**0.002**	**0.033**	0.276	0.986	**$5.42x10^{-4}$**	0.229	**0.006**	**0.025**	0.467	**$1.47x10^{-6}$**	0.987	0.841	0.102	0.556	0.327
6	$CD3^+$ T cells					**	–	**0.022**	0.150	0.500	**0.043**	0.108	**$1.12x10^{-4}$**	**0.013**	0.214	**0.002**	0.627	0.602	0.364	0.907	0.093
7	$CD8^+$ T cells	*	*			*	*	–	0.229	0.851	**0.033**	**$2.04x10^{-4}$**	0.090	0.425	0.054	0.074	0.365	0.614	0.258	0.556	0.258
8	$CD8^+$ naïve								–	0.694	0.511	0.489	0.229	0.128	0.907	0.260	0.701	0.907	0.413	0.467	0.374
9	$CD8^+$ central memory									–	0.694	0.878	0.202	0.231	0.932	0.932	0.280	0.096	0.057	0.986	0.771
10	$CD8^+$ effector memory					**	*	*			–	0.174	0.128	0.276	0.244	**$5.42x10^{-4}$**	0.777	0.973	0.056	0.777	0.291
11	$CD8^+$ terminal effector	*	*		*			**				–	0.293	0.855	**0.005**	0.328	0.214	0.947	0.266	0.603	0.235
12	$CD4^+$ T cells					**	**						–	**0.001**	0.446	**0.009**	0.907	0.510	0.763	0.855	0.199
13	$CD4^+$ naïve					*	*						**	–	0.987	**0.025**	0.174	0.544	0.532	0.511	0.675
14	$CD4^+$ central memory											**			–	0.446	0.328	0.688	0.354	0.777	0.228
15	$CD4^+$ effector memory					**	**				**		**	*		–	0.960	0.934	0.077	0.726	0.228
16	$CD4^+$ terminal effector																–	0.763	0.894	0.150	0.056
17	Tregs																	–	0.269	0.318	0.649
18	Th1-like		**																–	0.185	0.821
19	Th2-like																			–	0.111
20	Th17-like																				–
21	γ δ T cells	**																			
22	MAIT/NKT cells																				
23	B cells		*			**					*					*			*		
24	B naïve		*			**					*					*			*		
25	B memory		*			*		**			**	*				*					
26	Plasmablasts										*	*			**						
27	NK cells				**																
28	NK early																				
29	NK late				*																
30	Monocytes									*											
31	Monocytes classical																				
32	Monocytes transitional					*	*						*	*		*					
33	Monocytes nonclassical					*	**						**	**		*					
34	Dendritic cells		*												*				*		
35	pDCs																	**			
36	mDCs		*												*				*		
37	Granulocytes					**	**	*			**		*	*		**			*		
38	Neutrophils					**	**	*			**		**			**					
39	Basophils				*									*							
40	Eosinophils																				
41	$CD66b^-$ neutrophils								*												

		21	22	23	24	25	26	27	28	29	30	31	32	33	34	35	36	37	38	39	40	41
1	ACE	**1.12x10^{-4}**	0.881	0.603	0.603	0.162	0.613	0.726	0.328	0.881	0.511	0.310	0.385	0.128	0.532	0.392	0.425	0.260	0.162	0.701	0.854	0.960
2	sIL-2R	0.074	0.533	**0.029**	**0.029**	**0.022**	0.072	0.726	0.987	0.960	0.803	0.934	0.054	0.174	**0.032**	0.841	**0.043**	0.082	0.067	0.855	0.841	0.533
3	ESR (1 h)	0.603	0.987	0.751	0.751	0.405	0.687	0.533	0.987	0.489	0.907	0.960	0.425	0.726	0.291	0.776	0.310	0.960	0.907	0.651	0.724	0.108
4	CD4/CD8	0.293	0.651	0.726	0.726	0.385	0.432	**0.005**	0.174	**0.029**	0.726	0.777	0.365	0.328	0.881	0.763	0.987	0.907	0.960	**0.033**	0.776	0.328
5	Lymphocytes	0.229	0.425	**0.008**	**0.008**	**0.011**	0.153	0.328	0.829	0.150	0.751	0.701	**0.029**	**0.029**	0.250	0.789	0.187	**7.32x 10^{-6}**	**5.48x10^{-5}**	0.174	0.475	0.907
6	CD3^{+} T cells	0.174	0.365	0.260	0.260	0.060	0.334	0.310	0.276	0.162	0.676	0.533	**0.038**	**0.002**	0.228	0.776	0.117	**8.14x10^{-4}**	**2.04x10^{-4}**	0.128	0.763	0.960
7	CD8^{+} T cells	0.098	0.556	0.293	0.293	**0.005**	0.087	0.580	0.881	0.881	0.405	0.328	0.310	0.276	0.185	0.776	0.174	**0.043**	**0.025**	0.881	0.475	0.651
8	CD8^{+} naïve	0.214	0.054	0.777	0.777	0.987	0.763	0.651	0.580	0.511	0.150	0.090	0.627	0.187	0.841	0.674	0.829	0.244	0.310	0.511	0.841	**0.048**
9	CD8^{+} central memory	0.986	0.798	0.456	0.456	0.824	0.567	0.719	0.280	0.851	**0.046**	0.058	0.618	0.824	0.569	0.236	0.643	0.798	0.851	0.413	0.070	0.745
10	CD8^{+} effector memory	0.214	0.425	**0.011**	**0.011**	**0.008**	**0.036**	0.580	0.907	0.328	0.556	0.603	0.276	0.244	0.275	0.933	0.244	**8.14x10^{-4}**	**0.002**	0.405	0.382	0.777
11	CD8^{+} terminal effector	0.162	0.556	0.556	0.556	**0.019**	**0.043**	0.489	0.907	0.726	0.676	0.511	0.405	0.425	0.066	0.828	0.067	0.200	0.117	0.987	0.662	0.467
12	CD4^{+} T cells	0.310	0.627	0.229	0.229	0.074	0.542	0.138	0.214	0.098	0.627	0.489	**0.022**	**0.004**	0.393	0.486	0.244	**0.011**	**0.008**	0.067	0.402	0.934
13	CD4^{+} naïve	0.260	0.365	0.128	0.128	0.310	0.933	0.074	0.090	0.090	0.446	0.293	**0.029**	**0.002**	0.444	0.625	0.328	**0.043**	0.054	**0.025**	0.325	0.425
14	CD4^{+} central memory	0.533	0.347	0.726	0.726	0.054	**0.004**	0.907	0.310	0.855	0.701	0.580	0.701	0.533	**0.014**	0.298	**0.011**	0.310	0.187	0.385	0.486	0.651
15	CD4^{+} effector memory	0.229	0.293	**0.016**	**0.016**	**0.038**	0.171	0.214	0.603	0.082	0.777	0.726	**0.043**	**0.016**	0.243	※	0.162	**9.31x10^{-8}**	**7.32x10^{-6}**	0.117	0.637	0.701
16	CD4^{+} terminal effector	0.960	0.533	0.347	0.347	0.829	0.699	0.446	0.365	0.881	0.187	0.162	0.987	0.829	0.920	0.960	0.934	0.751	0.580	0.229	0.072	0.229
17	Tregs	0.132	0.111	0.675	0.675	0.675	0.271	0.243	0.881	0.160	0.243	0.266	0.738	0.776	0.241	**0.001**	0.318	0.934	0.960	0.345	0.594	0.498
18	Th1-like	0.291	0.056	**0.023**	**0.023**	0.250	0.071	0.789	0.854	0.498	0.802	0.973	0.185	0.213	**0.024**	0.137	**0.035**	**0.0498**	0.069	0.413	0.834	0.688
19	Th2-like	0.556	0.082	0.244	0.244	0.511	0.273	0.934	0.829	0.881	0.726	0.651	0.533	0.855	0.077	0.226	0.162	0.651	0.777	0.229	0.412	0.987
20	Th17-like	0.354	0.725	0.973	0.973	0.555	0.570	0.243	0.434	0.097	0.881	0.987	0.455	0.235	0.828	0.762	0.614	0.185	0.132	0.725	0.667	0.510
21	γδ T cells	–	0.934	0.385	0.385	0.260	0.894	0.726	0.385	0.829	0.244	0.128	0.467	0.128	0.751	0.325	0.676	0.244	0.200	0.726	0.662	0.603
22	MAIT/NKT cells		–	0.511	0.511	0.934	0.241	0.310	0.276	0.276	0.425	0.328	0.726	0.310	0.059	**0.006**	0.074	0.229	0.293	**0.048**	0.815	0.082
23	B cells			–	#	**0.029**	0.092	0.328	0.987	0.229	0.987	0.934	**0.048**	0.200	0.160	0.880	0.187	**0.025**	**0.048**	0.229	0.273	0.855
24	B naïve			#	–	**0.029**	0.092	0.328	0.987	0.229	0.987	0.934	**0.048**	0.200	0.160	0.880	0.187	**0.025**	**0.048**	0.229	0.273	0.855
25	B memory			*	*	–	**0.010**	0.907	0.987	0.751	0.651	0.603	0.150	0.310	0.102	0.662	0.098	**0.033**	**0.022**	0.467	0.130	0.405
26	Plasmablasts					*	–	0.674	0.841	0.554	0.933	0.907	0.464	0.699	**0.005**	0.165	**0.009**	0.120	0.100	0.233	0.300	0.724
27	NK cells							–	0.067	**2.28x10^{-5}**	0.881	0.987	0.187	0.128	0.544	0.372	0.425	0.276	0.328	**0.005**	0.531	0.150
28	NK early								–	0.187	0.467	0.260	0.467	0.054	0.266	0.577	0.174	0.627	0.533	**0.038**	0.519	0.276
29	NK late							**		–	0.580	0.676	0.082	0.074	0.476	0.334	0.347	0.098	0.128	**0.016**	0.907	0.229
30	Monocytes										–	**1.47x10^{-6}**	0.244	0.934	0.802	0.737	0.726	0.907	0.960	0.726	**0.021**	0.090
31	Monocytes classical										**	–	0.446	0.751	0.947	0.802	0.987	0.829	0.855	0.603	**0.026**	0.082
32	Monocytes transitional			*	*								–	**0.004**	0.132	0.894	0.082	**0.033**	**0.029**	0.162	0.841	0.556
33	Monocytes nonclassical												**	–	0.172	0.947	0.082	**0.013**	**0.009**	0.067	0.920	0.751
34	Dendritic cells						**								–	0.078	**2.20x10^{-7}**	0.148	0.116	0.069	0.692	0.738
35	pDCs		**													–	0.115	0.841	0.907	0.197	0.723	0.298
36	mDCs						**								**		–	0.090	0.060	**0.048**	0.815	0.701
37	Granulocytes			*	*	*							*	*				–	**9.31x 10^{-8}**	0.128	0.802	0.829
38	Neutrophils			*	*	*							*	**				**	–	0.150	0.854	0.987
39	Basophils		*					**	*	*							*			–	0.298	0.347
40	Eosinophils										*	*									–	0.422
41	CD66b^{-} neutrophils																					–

Numerical data are *p* values computed by Spearman's rank correlation test. Blue box indicates positive correlation. Red box denotes negative correlation. Abbreviations are the same as those in Supplementary Table S1. *$p < 0.05$, **$p < 0.01$. #; Cannot calculate due to

absolute value of correlation coefficient being 1. ACE; angiotensin-converting enzyme, ESR; erythrocyte sedimentation rate, sIL-2R; soluble interleukin-2 receptor.

Supplementary Table S3. Correlation matrix of serum ACE level, serum sIL-2R level, ESR, CD4/CD8 ratio and leukocyte subset proportions in the acute phase of Vogt-Koyanagi-Harada disease patients.

		1	2	3	4	5	6	7	8	9	10	11	12	13	14	15	16	17	18	19	20
1	ACE	–	0.747	※	0.819	0.879	※	0.645	0.702	**0.021**	0.819	0.819	0.645	※	0.819	0.760	※	0.760	0.148	0.758	0.939
2	sIL-2R		–	0.391	0.188	**0.037**	0.188	0.624	0.747	0.391	0.873	0.505	0.104	0.104	**0.037**	0.285	0.505	**0.037**	0.391	0.054	0.505
3	ESR (1 h)			–	0.699	0.058	0.129	0.310	0.848	0.938	0.969	0.210	0.248	0.175	0.758	0.818	0.558	0.788	0.848	0.938	0.758
4	CD4/CD8				–	0.294	0.482	**0.023**	0.939	0.427	0.432	**0.023**	0.180	0.148	0.094	0.294	0.760	0.119	0.760	0.403	0.939
5	Lymphocytes		*			–	**0.014**	0.939	0.589	0.613	0.879	0.939	**0.014**	**0.003**	0.180	0.383	0.482	0.337	0.589	0.558	0.432
6	$CD3^+$ T cells					*	–	0.589	0.215	0.728	0.535	0.702	**0.003**	**0.036**	0.119	0.094	0.094	0.119	0.939	0.788	0.071
7	$CD8^+$ T cells				*			–	0.482	0.452	0.148	**0.003**	0.879	0.645	0.535	※	0.148	0.702	0.760	0.268	0.294
8	$CD8^+$ naïve								–	0.333	0.819	※	0.294	0.337	0.879	0.180	0.432	0.432	0.879	0.192	0.253
9	$CD8^+$ central memory	*								–	0.613	0.788	0.427	0.878	0.175	0.558	0.699	0.670	0.159	0.448	0.788
10	$CD8^+$ effector memory										–	0.180	0.879	0.482	0.535	0.215	**0.007**	0.383	0.383	0.818	**0.023**
11	$CD8^+$ terminal effector				*			**				–	0.819	0.535	0.702	0.702	0.215	0.535	0.535	0.379	0.482
12	$CD4^+$ T cells					*	**						–	**0.014**	0.071	0.094	0.294	0.119	0.760	0.878	0.215
13	$CD4^+$ naïve					**	*						*	–	0.294	0.383	0.819	0.337	0.482	0.758	0.645
14	$CD4^+$ central memory		*												–	0.119	0.253	**0.023**	0.645	0.613	0.215
15	$CD4^+$ effector memory															–	0.071	**0.007**	0.702	0.908	**0.014**
16	$CD4^+$ terminal effector										**						–	0.148	0.535	0.699	**$4.54x10^{-4}$**
17	Tregs		*												*	**		–	※	0.848	0.052
18	Th1-like																		–	0.248	0.702
19	Th2-like																			–	0.758
20	Th17-like										*					*	**				–
21	γ δ T cells						*										*				
22	MAIT/NKT cells		*																		
23	B cells		*			*							*								
24	B naïve		*			*								*							
25	B memory				*					*											
26	Plasmablasts		*																	**	
27	NK cells						*														
28	NK early																				*
29	NK late																				
30	Monocytes																				
31	Monocytes classical																				
32	Monocytes transitional																				
33	Monocytes nonclassical																				
34	Dendritic cells																				
35	pDCs	*								*											
36	mDCs																			*	
37	Granulocytes		*			**								*							
38	Neutrophils		*			#	*						*	**							
39	Basophils															*					
40	Eosinophils											*									
41	$CD66b^-$ neutrophils																			*	

		21	22	23	24	25	26	27	28	29	30	31	32	33	34	35	36	37	38	39	40	41
1	ACE	0.535	0.589	0.253	0.294	0.215	0.908	0.702	0.535	0.939	0.702	0.702	0.337	0.129	0.482	**0.049**	0.645	0.702	0.879	0.432	0.310	0.819
2	sIL-2R	0.285	**0.037**	**0.037**	**0.037**	0.188	**0.037**	0.285	0.188	0.624	0.624	0.624	0.873	0.805	0.285	0.188	0.104	**0.037**	**0.037**	0.747	0.493	0.188
3	ESR (1 h)	0.090	※	0.427	0.310	0.939	0.892	0.090	0.355	0.090	0.129	0.129	0.248	0.215	0.558	0.816	0.848	0.090	0.058	0.289	0.139	0.531
4	CD4/CD8	0.879	0.094	0.094	0.180	**0.014**	0.192	0.819	0.760	0.383	0.337	0.337	0.819	0.403	0.094	0.090	0.119	0.337	0.294	0.215	0.144	0.645
5	Lymphocytes	0.119	0.180	**0.023**	**0.014**	0.215	0.310	0.071	0.094	0.253	0.702	0.702	0.645	0.758	0.589	0.229	0.337	**4.54×10^{-4}**	**#**	0.939	0.939	0.432
6	$CD3^+$ T cells	**0.014**	0.337	0.094	0.119	0.383	0.788	**0.036**	0.052	0.337	0.939	0.939	0.760	0.878	0.819	0.403	0.535	0.052	**0.014**	0.482	0.758	※
7	$CD8^+$ T cells	0.215	0.337	0.432	0.535	0.094	0.248	0.215	0.535	0.148	0.482	0.482	0.760	0.504	0.148	0.192	0.294	0.879	0.939	0.535	0.090	0.589
8	$CD8^+$ naïve	0.383	0.819	0.879	0.819	0.819	0.229	0.432	0.432	※	0.760	0.760	0.482	0.818	0.337	0.818	0.482	0.760	0.589	0.432	0.939	0.071
9	$CD8^+$ central memory	0.452	0.210	0.102	0.210	**0.049**	0.339	0.670	0.452	0.908	0.699	0.699	0.641	0.117	0.090	**0.014**	0.159	0.504	0.613	0.310	0.328	0.558
10	$CD8^+$ effector memory	0.215	0.645	0.939	0.939	0.879	0.939	0.215	0.180	0.294	0.337	0.337	0.148	0.355	0.702	0.848	0.589	0.939	0.879	0.383	0.969	0.879
11	$CD8^+$ terminal effector	0.215	0.337	0.482	0.535	0.148	0.403	0.294	0.702	0.148	0.432	0.432	0.939	0.452	0.253	0.310	0.383	0.879	0.939	0.383	**0.041**	0.939
12	$CD4^+$ T cells	0.052	0.253	**0.023**	0.052	0.119	0.699	0.148	0.119	0.702	0.939	0.939	※	0.585	0.589	0.115	0.432	0.052	**0.014**	0.294	0.878	0.939
13	$CD4^+$ naïve	0.294	0.294	0.052	**0.036**	0.215	0.504	0.253	0.253	0.645	0.645	0.645	0.645	0.788	0.760	0.248	0.535	**0.014**	**0.003**	0.819	0.908	0.760
14	$CD4^+$ central memory	0.294	0.071	0.071	0.215	0.052	0.192	0.482	0.294	0.939	0.215	0.215	0.482	0.355	0.071	0.144	0.052	0.253	0.180	0.180	0.613	0.432
15	$CD4^+$ effector memory	0.215	0.148	0.180	0.294	0.215	0.788	0.253	0.071	0.879	0.215	0.215	0.119	0.102	0.337	0.355	0.253	0.432	0.383	**0.014**	0.310	0.589
16	$CD4^+$ terminal effector	**0.036**	0.482	0.535	0.645	0.879	※	0.052	0.052	0.215	0.482	0.482	0.180	0.504	0.760	0.939	0.535	0.535	0.482	0.337	0.788	0.879
17	Tregs	0.432	0.094	0.215	0.383	0.180	0.427	0.482	0.215	0.939	0.071	0.071	0.071	0.268	0.180	0.452	0.119	0.432	0.337	0.052	0.403	0.939
18	Th1-like	0.383	0.645	0.939	0.760	0.760	0.355	0.939	0.939	0.589	0.939	0.939	0.939	0.144	※	0.452	0.760	0.482	0.589	0.294	0.908	0.337
19	Th2-like	0.558	0.058	0.310	0.229	0.268	**0.003**	0.818	0.558	0.478	0.452	0.452	0.818	0.969	0.058	0.448	**0.041**	0.289	0.558	0.788	0.215	**0.021**
20	Th17-like	0.071	0.337	0.432	0.535	0.702	0.939	0.071	**0.036**	0.337	0.337	0.337	0.094	0.379	0.645	0.848	0.432	0.482	0.432	0.180	0.908	0.760
21	γδ T cells	–	0.645	0.215	0.253	0.645	0.848	**0.014**	0.052	0.180	0.645	0.645	※	0.728	0.939	0.452	0.819	0.180	0.119	0.589	0.531	0.819
22	MAIT/NKT cells		–	**0.023**	**0.036**	**0.023**	**0.016**	0.337	0.094	0.535	0.180	0.180	0.589	0.355	**0.007**	0.115	**4.54×10^{-4}**	0.094	0.180	0.294	0.102	0.215
23	B cells		*	–	**4.54×10^{-4}**	**0.007**	0.159	0.180	0.071	0.535	0.819	0.819	0.760	0.403	0.119	**0.008**	0.071	**0.014**	**0.023**	0.337	0.310	0.482
24	B naïve		*	**	–	**0.036**	0.159	0.119	0.052	0.337	0.939	0.939	0.589	0.641	0.215	**0.033**	0.119	**0.003**	**0.014**	0.589	0.355	0.432
25	B memory		*	**	*	–	0.129	0.702	0.337	0.819	0.432	0.432	0.939	0.159	**0.023**	**0.002**	**0.036**	0.180	0.215	0.148	0.090	0.535
26	Plasmablasts		*				–	0.699	0.452	0.531	0.289	0.289	0.969	0.954	**0.016**	0.317	**0.006**	0.175	0.310	0.969	0.306	**0.008**
27	NK cells	*						–	**0.003**	**0.014**	0.760	0.760	0.939	※	0.879	0.613	0.535	0.052	0.071	0.879	0.788	0.760
28	NK early							**	–	0.071	0.760	0.760	0.645	0.531	0.432	0.355	0.215	0.052	0.094	0.432	0.613	0.760
29	NK late							*		–	0.645	0.645	0.819	0.478	※	0.908	0.645	0.148	0.253	0.432	0.558	0.337
30	Monocytes										–	#	**0.023**	0.229	0.071	0.878	0.094	0.760	0.702	0.148	0.192	0.645
31	Monocytes classical										#	–	**0.023**	0.229	0.071	0.878	0.094	0.760	0.702	0.148	0.192	0.645
32	Monocytes transitional										*	*	–	0.403	0.589	0.531	0.535	0.589	0.645	0.180	0.613	0.702
33	Monocytes nonclassical													–	0.210	0.162	0.355	0.818	0.758	**0.003**	0.074	0.452
34	Dendritic cells		**			*	*								–	0.115	**4.54×10^{-4}**	0.432	0.589	0.253	0.068	0.180
35	pDCs			**	*	**										–	0.159	0.192	0.229	0.229	0.188	0.728
36	mDCs		**			*	**								**		–	0.215	0.337	0.337	0.129	0.119
37	Granulocytes			*	**													–	**4.54×10^{-4}**	※	0.818	0.294
38	Neutrophils			*	*													**	–	0.939	0.939	0.432
39	Basophils													**						–	0.129	0.383
40	Eosinophils																				–	0.939
41	$CD66b^-$ neutrophils						**															–

Footnotes are the same as those in Supplementary Table S2. ※; Cannot calculate due to correlation coefficient being less than 1×10^{-10}.

Supplementary Table S4. Correlation matrix of CD4/CD8 ratio and leukocyte subset proportions in healthy controls.

		1	2	3	4	5	6	7	8	9	10	11	12	13	14	15	16	17	18
1	**CD4/CD8**	–	0.570	0.120	**0.021**	0.823	0.911	0.610	0.086	0.352	0.233	0.260	0.670	0.570	0.435	0.736	0.139	0.456	0.693
2	**Lymphocytes**		–	**0.015**	**0.037**	0.233	0.456	**0.028**	0.493	0.183	0.911	**0.010**	0.091	0.823	0.062	**0.007**	0.120	**0.047**	**0.047**
3	**CD3^{+} T cells**		*	–	**2.60x10^{-4}**	0.139	0.779	0.183	0.651	0.289	0.867	0.071	0.157	0.823	0.157	0.207	0.102	**0.037**	0.183
4	**CD8^{+} T cells**	*	*	**	–	0.207	0.823	0.207	0.320	0.610	0.823	**0.047**	0.435	0.955	0.126	0.352	**0.047**	0.086	0.183
5	**CD8^{+} naïve**					–	0.823	0.736	0.352	**0.047**	0.071	0.867	0.756	0.531	0.866	0.233	0.385	0.867	**0.047**
6	**CD8^{+} central memory**						–	0.736	0.456	0.531	0.160	0.570	0.978	**0.015**	0.778	0.456	0.385	0.867	0.651
7	**CD8^{+} effector memory**		*					–	0.183	0.651	0.531	**0.004**	0.062	0.911	**0.031**	0.120	0.456	**0.010**	0.493
8	**CD8^{+} terminal effector**								–	0.102	**0.010**	0.086	0.629	0.086	0.317	0.867	0.207	0.651	0.736
9	**CD4^{+} T cells**					*				–	**0.007**	0.779	0.076	0.102	0.629	0.102	0.955	0.352	0.233
10	**CD4^{+} naïve**								*	**	–	0.420	0.490	**0.028**	0.691	0.570	0.456	0.955	0.531
11	**CD4^{+} central memory**		*		*			**				–	0.243	0.693	**0.002**	0.183	**0.047**	0.058	0.352
12	**CD4^{+} effector memory**												–	0.301	0.254	0.157	0.978	**0.011**	0.778
13	**CD4^{+} terminal effector**						*				*			–	0.844	0.736	0.456	0.610	0.610
14	**Tregs**							*				**			–	0.417	0.076	0.083	0.670
15	**Th1-like**		**													–	0.610	0.207	**0.010**
16	**Th2-like**				*							*					–	0.651	0.420
17	**Th17-like**		*	*				*					*					–	0.570
18	**$\gamma\delta$ T cells**		*			*										*			–
19	**MAIT/NKT cells**																		
20	**B cells**		*					**				*							
21	**B naïve**							*								*			
22	**B memory**		*					*				**			**				
23	**Plasmablasts**							**	*			*							
24	**NK cells**												*						
25	**NK early**												*						
26	**NK late**												*						
27	**Monocytes**							*							*			*	
28	**Monocytes classical**														*			*	
29	**Monocytes transitional**																		
30	**Monocytes nonclassical**	**																	
31	**Dendritic cells**																		
32	**pDCs**																		
33	**mDCs**																		
34	**Granulocytes**		#	*	*			*				*				**		*	*
35	**Neutrophils**		#	*	*			*				*				**		*	*
36	**Basophils**																		
37	**Eosinophils**																		
38	**CD66b^{-} neutrophils**								*			*			*				

		19	20	21	22	23	24	25	26	27	28	29	30	31	32	33	34	35	36	37	38
1	CD4/CD8	0.844	0.352	0.570	0.823	0.213	0.823	0.867	0.823	0.736	0.610	0.058	**0.007**	0.823	0.978	0.823	0.570	0.570	0.230	0.289	0.233
2	Lymphocytes	0.435	**0.037**	0.071	**0.021**	0.060	0.456	0.233	0.456	0.385	0.420	0.867	0.866	0.911	0.778	0.911	#	#	0.471	0.911	0.320
3	CD3$^+$ T cells	0.608	0.183	0.385	0.207	0.177	0.456	0.260	0.456	0.493	0.385	0.289	0.588	0.779	0.713	※	**0.015**	**0.015**	0.866	0.823	0.531
4	CD8$^+$ T cells	0.713	0.139	0.289	0.233	0.105	0.693	0.456	0.693	0.493	0.385	0.120	0.243	0.823	0.734	0.955	**0.037**	**0.037**	0.691	0.610	0.260
5	CD8$^+$ naïve	0.888	0.955	0.823	0.823	0.910	0.610	0.867	0.610	0.531	0.693	0.289	0.490	0.823	0.333	0.610	0.233	0.233	0.608	0.352	0.779
6	CD8$^+$ central memory	0.713	0.823	0.955	0.736	0.887	0.911	0.955	0.911	0.651	0.610	0.160	0.821	0.385	0.076	0.911	0.456	0.456	0.548	0.207	0.779
7	CD8$^+$ effector memory	0.146	**0.004**	**0.037**	**0.015**	**0.007**	0.183	0.207	0.183	**0.037**	0.086	0.867	0.670	0.867	0.933	0.823	**0.028**	**0.028**	0.108	0.420	0.102
8	CD8$^+$ terminal effector	0.799	0.058	0.120	0.320	**0.033**	0.823	0.823	0.823	0.610	0.736	0.651	0.076	0.867	0.778	0.610	0.493	0.493	0.866	0.183	**0.047**
9	CD4$^+$ T cells	0.333	0.867	0.736	0.456	0.977	0.531	0.289	0.531	0.693	0.651	0.955	0.117	0.823	0.317	0.651	0.183	0.183	0.301	0.420	0.693
10	CD4$^+$ naïve	0.608	0.456	0.736	0.779	0.379	0.955	0.779	0.955	0.867	0.955	0.736	0.126	0.736	0.192	0.651	0.911	0.911	0.588	0.420	0.385
11	CD4$^+$ central memory	0.333	**0.015**	0.071	**0.002**	**0.015**	0.260	0.160	0.260	0.102	0.120	0.610	0.365	0.736	0.821	0.693	**0.010**	**0.010**	0.317	0.456	**0.021**
12	CD4$^+$ effector memory	0.143	0.301	0.568	0.146	0.411	**0.035**	**0.040**	**0.035**	0.146	0.168	0.568	0.459	0.821	0.820	0.888	0.091	0.091	0.119	0.933	※
13	CD4$^+$ terminal effector	0.243	0.570	0.570	※	0.565	0.352	0.320	0.352	0.352	0.260	0.420	0.756	0.911	0.349	0.570	0.823	0.823	0.286	0.736	0.867
14	Tregs	0.088	0.180	0.317	**0.006**	0.168	0.091	0.069	0.091	**0.020**	**0.014**	0.548	0.329	0.230	0.487	0.168	0.062	0.062	0.154	0.713	**0.020**
15	Th1-like	0.568	0.086	**0.047**	0.160	0.154	0.955	0.693	0.955	0.779	0.955	0.570	0.243	0.570	0.471	0.736	**0.007**	**0.007**	0.435	0.493	0.779
16	Th2-like	0.799	0.420	0.610	0.086	0.396	0.651	0.289	0.651	0.823	0.651	0.260	0.317	0.385	0.509	0.320	0.120	0.120	0.778	0.779	0.139
17	Th17-like	0.062	0.086	0.260	0.160	0.088	0.102	0.183	0.102	**0.037**	**0.047**	0.955	0.670	0.867	0.933	0.867	**0.047**	**0.047**	0.317	0.736	0.456
18	γδ T cells	※	0.183	0.058	0.493	0.213	0.320	0.651	0.320	0.736	0.651	0.693	0.588	0.456	0.257	0.779	**0.047**	**0.047**	0.844	0.493	0.693
19	MAIT/NKT cells	–	0.588	0.608	0.365	0.563	0.117	0.333	0.117	**0.002**	**0.005**	0.910	0.787	0.382	0.733	0.301	0.435	0.435	0.051	0.756	0.382
20	B cells		–	**8.63x10^{-4}**	0.071	**4.26x10^{-6}**	0.823	0.693	0.823	0.289	0.456	0.823	0.608	0.320	0.417	0.352	**0.037**	**0.037**	0.435	0.260	0.102
21	B naïve		**	–	0.160	**0.001**	0.693	0.867	0.693	0.456	0.693	0.823	0.844	0.289	0.243	0.420	0.071	0.071	0.435	0.493	0.120
22	B memory				–	0.105	0.139	**0.037**	0.139	0.139	0.160	0.867	0.866	0.610	0.844	0.493	**0.021**	**0.021**	0.091	0.531	0.058
23	Plasmablasts		**	**		–	0.887	0.798	0.887	0.254	0.396	0.647	0.393	0.346	0.438	0.379	0.060	0.060	0.533	0.213	0.066
24	NK cells						–	**0.002**	#	**0.047**	**0.028**	0.693	0.888	0.260	0.272	0.352	0.456	0.456	0.108	0.779	0.651
25	NK early				*		**	–	**0.002**	0.160	0.102	0.955	0.933	0.385	0.548	0.320	0.233	0.233	0.136	0.651	0.531
26	NK late						#	**	–	**0.047**	**0.028**	0.693	0.888	0.260	0.272	0.352	0.456	0.456	0.108	0.779	0.651
27	Monocytes	**					*		*	–	**3.31x10^{-5}**	0.823	0.349	0.456	0.691	0.420	0.385	0.385	**0.040**	0.531	0.102
28	Monocytes classical	**					*		*	**	–	0.610	0.272	0.320	0.588	0.260	0.420	0.420	0.083	0.570	0.120
29	Monocytes transitional											–	0.099	0.823	0.365	0.651	0.867	0.867	0.548	0.260	0.233
30	Monocytes nonclassical												–	0.670	0.616	0.756	0.866	0.866	0.596	0.180	0.091
31	Dendritic cells													–	**0.007**	**0.002**	0.911	0.911	0.629	0.289	0.610
32	pDCs													**	–	0.157	0.778	0.778	0.977	0.382	0.955
33	mDCs													**		–	0.911	0.911	0.490	0.352	0.456
34	Granulocytes		*		*												–	#	0.471	0.911	0.320
35	Neutrophils		*		*												#	–	0.471	0.911	0.320
36	Basophils									*									–	0.756	0.317
37	Eosinophils																			–	0.207
38	CD66b$^-$ neutrophils																				–

Footnotes are the same as those in Supplementary Table S2 and Supplementary Table S3.
